# Single-Cell RNA-Seq of Pituitary and Ovary Identifies Regulators of Reproduction in Yellow Catfish (*Pelteobagrus fulvidraco*)

**DOI:** 10.3390/ani16132044

**Published:** 2026-07-02

**Authors:** Yuanqi Guo, Zhaoxian Li, Mengjie Chen, Ji Chen, Binbin Tao, Hongrui Luo, Jie Mei, Yang Xiong, Wei Hu, Yanlong Song

**Affiliations:** 1 State Key Laboratory of Breeding Biotechnology and Sustainable Aquaculture, Hubei Hongshan Laboratory, Institute of Hydrobiology, Chinese Academy of Sciences, Wuhan 430072, China; guoyuanqi@ihb.ac.cn (Y.G.); lizhaoxian@ihb.ac.cn (Z.L.); 18872084600@163.com (M.C.); chenji@ihb.ac.cn (J.C.); taobinb@ihb.ac.cn (B.T.); hrluo90@163.com (H.L.); meijie@ihb.ac.cn (J.M.); xiongyang@ihb.ac.cn (Y.X.); huwei@ihb.ac.cn (W.H.); 2 College of Advanced Agricultural Sciences, University of Chinese Academy of Sciences, Beijing 100049, China; 3 Key Laboratory of Exploration and Utilization of Aquatic Genetic Resources, Ministry of Education, Shanghai Ocean University, Shanghai 201306, China; 4 Key Laboratory of Freshwater Aquatic Genetic Resources, Ministry of Agriculture and Rural Affairs, Shanghai Ocean University, Shanghai 201306, China

**Keywords:** *Pelteobagrus fulvidraco*, pituitary, ovary, single-cell sequencing, reproductive regulation

## Abstract

The pituitary and gonads serve as central regulatory hubs and functional organs for gametogenesis and maturation. In this study, we performed single-cell RNA sequencing (scRNA-seq) of the pituitary and ovary in pre-spawning *Pelteobagrus fulvidraco* to elucidate the cellular landscape and regulatory pathways governing gonadal development and oocyte maturation. Pituitaries from four female fish (weight: 43.8 ± 3.2 g; length: 14.1 ± 0.7 cm) and four male fish (weight: 78.2 ± 11.2 g; length: 18.9 ± 1.1 cm) were subjected to single-cell transcriptomic analysis. A total of 17 distinct cell types were identified in the female pituitary, whereas 15 cell types were detected in the male pituitary. Both male and female pituitaries comprised multiple hormone-secreting endocrine populations, indicating a largely conserved cellular composition. However, sex-specific differences were observed in thyrotrope subtypes, suggesting potential sexual dimorphism in pituitary endocrine regulation. Examination of receptor gene expression revealed cell-type-specific regulatory capacities, highlighting gonadotropin, steroid, and neuropeptide responsiveness across pituitary populations. In the ovary, 10 cell types were identified, with granulosa cells (~22.9%) and theca cells (~8.6%) showing distinct transcriptional profiles. Follicle-stimulating hormone receptor (*fshr*) was highly expressed in granulosa cells, whereas luteinizing hormone receptor (*lhcgr*) and steroidogenic genes (*hsd3b1*, *pgr*) were predominantly localized in theca cells, indicating functional compartmentalization of gonadotropin and steroid signaling. Prostaglandin (PG) and melatonin (MT) pathways were implicated in paracrine regulation: the prostaglandin synthase *ptgs2a* was expressed in theca, germ, and immune cells, while *ptger2a* was expressed in granulosa cells; melatonin synthesis genes (*aanat1*, *aanat2*, *asmtl*) were confined to granulosa cells, with receptors (*mtnr1ab*) in germ cells. These findings suggest that ovarian paracrine signaling complements systemic endocrine control to modulate oocyte maturation and ovulation. This single-cell atlas provides a high-resolution framework of reproductive cell types and signaling networks in *P. fulvidraco*, offering insights for improving artificial breeding and reproductive management in aquaculture.

## Simple Summary

This study provides the first single-cell transcriptomic atlas of the pituitary gland in male and female and ovary in pre-spawning *Pelteobagrus fulvidraco*. Distinct endocrine and non-endocrine cell populations were identified, revealing significant cellular heterogeneity and sex-specific characteristics in the pituitary. Cell-type-specific expression of hormone receptors suggested complex regulatory networks involving gonadotropins, steroid hormones, and neuropeptides. In the ovary, granulosa and theca cells exhibited distinct functional specializations, particularly in gonadotropin responsiveness and steroidogenesis. Furthermore, prostaglandin and melatonin signaling pathways were found to participate in local paracrine regulation of follicular development and oocyte maturation. These findings improve our understanding of the molecular and cellular mechanisms underlying reproduction in *P. fulvidraco* and provide valuable resources for the development of artificial breeding technologies and reproductive management strategies in aquaculture.

## 1. Introduction

Yellow catfish (*Pelteobagrus fulvidraco*) is an economically important freshwater aquaculture species widely cultivated in China due to its rapid growth rate, desirable flesh quality, and the absence of intermuscular spicules [1,2]. The annual production of cultured yellow catfish has reached 627,689 tons [3]. The rapid expansion of intensive aquaculture has led to a growing need for stable fry supply and efficient artificial breeding technologies. However, reproductive dysfunction under captive conditions, asynchronous gonadal development, low ovulation response, and inconsistent fertilization rates remain major constraints in commercial seed production [4,5]. Low spawning rates, together with high mortality of broodstock, are commonly observed during the artificial breeding of *P. fulvidraco* [6]. Therefore, it is imperative to identify the regulatory factors that govern reproduction in order to develop more efficient and safe methods of induced spawning and breeding.

In teleost, reproduction is primarily regulated by the hypothalamic–pituitary–gonadal (HPG) axis, which coordinates gonadal development, gametogenesis, final gametophyte maturation, ovulation, and spermiation through endocrine signaling [7,8]. The pituitary gland acts as a central endocrine organ by secreting gonadotropins, including follicle-stimulating hormone (FSH) and luteinizing hormone (LH), together with other hormones such as growth hormone (GH), prolactin (PRL), and thyroid-stimulating hormone (TSH), which directly or indirectly influence gonadal function [9,10]. In female fish, ovarian development depends on coordinated interactions among germ cells and multiple somatic cell types, including granulosa cells, theca cells, stromal cells, endothelial cells, and immune cells, which collectively regulate folliculogenesis and oocyte maturation [11,12]. Despite extensive research into the mechanisms of reproductive endocrine regulation in fish, the cell types involved in this regulation and their interrelationships remain unclear.

Single-cell RNA sequencing (scRNA-seq) has emerged as a transformative technology that enables transcriptomic profiling at single-cell resolution, facilitating the identification of novel cell populations, developmental trajectories, and cell–cell communication networks [13,14]. In fish ovaries, single-cell approaches have begun to uncover stage-specific germ cell differentiation pathways and somatic niche functions during follicle development [15,16]. Similarly, single-cell transcriptomics in zebrafish pituitary have identified endocrine cell plasticity and Gnrh3 may play a role in the specialization of endocrine cell types [17]. Simultaneous single-cell profiling of the pituitary and ovary in teleosts, especially in aquaculture-relevant species such as yellow catfish, provides a powerful approach to dissect reproductive regulatory networks and uncover key modulators of fertility.

In *P. fulvidraco*, previous research has mainly focused on sex determination, growth regulation, nutrition, and induced breeding technologies [6,18,19]. However, the cellular composition of the pituitary, sex-specific endocrine differences, ovarian microenvironment dynamics, and the molecular mechanisms governing final oocyte maturation remain poorly understood. This knowledge gap limits the rational design of spawning-inducing agents, many of which are still based on empirical hormone combinations and show variable efficiency under farming conditions [20]. Therefore, in the present study, we performed single-cell transcriptomic profiling of pituitary tissues from female and male yellow catfish, together with scRNA-seq of ovarian tissue. By integrating these datasets, we aimed to: (1) construct a high-resolution cellular atlas of the pituitary and ovary; (2) identify sex-biased endocrine populations and reproductive regulators; (3) reveal key signaling pathways controlling follicular development and oocyte maturation; and (4) screen candidate bioactive factors for reproductive induction.

## 2. Materials and Methods

### 2.1. Ethics

All procedures involving *P. fulvidraco* were approved by the Institutional Animal Care and Use Committee of the Institute of Hydrobiology, Chinese Academy of Sciences (Approval number: IHB/LL/2024078), and complied with national guidelines for the care and use of laboratory animals.

### 2.2. Fish Maintenance and Tissue Collection

Sexually mature *P. fulvidraco* were obtained from Fisheries Research institute, Wuhan Academy of Agricultural (Wuhan, China). Fish were maintained in aerated freshwater ponds (18 ± 1 °C) for at least 5 days prior to sampling. In late March, fish were euthanized using MS-222 (200 mg/L), and pituitary glands and ovaries were immediately dissected under sterile conditions. To meet the tissue input requirements for single-cell RNA sequencing, pituitaries from four female fish (body weight: 43.8 ± 3.2 g; total length: 14.1 ± 0.7 cm) or four male fish (body weight: 78.2 ± 11.2 g; total length: 18.9 ± 1.1 cm) were pooled into a single 1.5 mL Eppendorf tubes containing MACS Tissue Storage Solution (Beijing Yunpei Biotechnology Co., Ltd., Beijing, China) and kept on ice until processing for single-cell isolation.

### 2.3. Single-Cell Suspension Preparation

Dissected pituitaries were washed in ice-cold PBS (Hyclone, Logan, UT, USA, SH30256.01) and dissociated using SeekMate Tissue Dissociation Reagent Kit A Pro (SeekGene, Beijing, China, K01801301) from SeekGene as instructions. Ovarian tissue was dissociated into single cells using the same method, with the addition of 0.1% DNase I (Sigma, St. Louis, MO, USA, 9003-98-9) treatment. After erythrocyte removal using a red cell lysis solution (Solarbio, Beijing, China, R1010) according to the manufacturer’s instructions, cell concentration and viability were assessed using a Fluorescence Cell Analyzer (Countstar^®^ Rigel S2, Countstar, Shanghai, China) with AO/PI staining reagent. Only suspensions with >90% viable cells and <10% clustered cells were used for library construction. Finally fresh cells were washed twice in the RPMI1640 (Gibco, 11875119, Thermo Fisher Scientific Inc., Waltham, MA, USA) and then resuspended at 1 × 10^6^ cells per mL in RPMI1640 and 2% FBS (Gibco, 10100147C).

### 2.4. Library Preparation and scRNAseq

Single-cell cDNA library preparation and sequencing were performed by SeekGene BioSciences Co., Ltd. (Beijing, China). Single-cell RNA-Seq libraries were generated using the SeekOne^®^ Digital Droplet Single Cell 3′ library preparation kit (SeekGene, Catalog No. K00202). Briefly, an appropriate number of cells were mixed with reverse transcription reagents and loaded into the sample well of the SeekOne^®^ chip S3. Barcoded Hydrogel Beads (BHBs) and partitioning oil were then added to their respective wells in the chip. Following emulsion droplet generation, reverse transcription was carried out at 42 °C for 90 min and subsequently inactivated at 85 °C for 5 min. The resulting cDNA was purified from broken droplets and amplified via PCR. The amplified cDNA was further purified, fragmented, end-repaired, A-tailed, and ligated to sequencing adapters. Indexed PCR was then performed to amplify DNA representing the 3′ poly(A) regions of expressed genes, incorporating both cell barcodes and unique molecular identifiers (UMIs). The indexed libraries were cleaned using VAHTS DNA Clean Beads (Vazyme, Nanjing, China, N411-01), quantified with Qubit (Thermo Fisher Scientific, Q33226), and assessed for fragment size distribution using a Bio-Fragment Analyzer (Bioptic, Qsep400, New Taipei City, Taiwan, China). Finally, libraries were sequenced on an Illumina NovaSeq 6000 platform with paired-end 150 bp reads (Illumina, San Diego, CA, USA).

### 2.5. Data Processing and Statistical Analysis

The data presented were pooled results from four samples. Raw sequencing data were preprocessed using fastp v0.19.5 to remove adapter contamination and low-quality reads, thereby ensuring the accuracy of downstream analyses. The clean reads were then aligned to the *Tachysurus fulvidraco* genome: NCBI RefSeq assembly GCF_022655615.1 and annotation: NCBI *Tachysurus fulvidraco* Annotation Release 101 using Cell Ranger v6.1, which also performed UMI counting and generated the gene–cell expression matrix.

To accurately identify true cells, two complementary approaches were employed:

1. UMI-based filtering: The 99th percentile of the top 3000 UMI counts was defined as the maximum estimated total UMI (m). Barcodes with UMI counts exceeding m/10 were considered as captured cells.
2. RNA expression-based identification: Clustered integrated single-cell datasets were analyzed and visualized using Seurat 4.1.1, with the clustering resolution set to 0.2.

Data obtained from both approaches were merged to generate a comprehensive dataset for downstream analyses. Quality control metrics, including nFeature_RNA, nCount_RNA, and the proportion of mitochondrial gene expression (percent.mt), were calculated for each cell. To further evaluate data quality, Feature plots of these metrics were generated and projected onto the UMAP embedding, allowing visualization of their distribution across all cells and transcriptionally defined clusters. This approach was used to assess the quality status of individual cell clusters and to identify potential low-quality or stressed cell populations. Cell type annotation was performed using SingleR, based on both its built-in reference datasets and SeekGene’s custom tissue reference sets. Single-cell visualizations were generated using the SeekGeneOnline cloud platform (seeksoul). To ensure annotation accuracy, all predicted cell identities were further validated through manual curation based on canonical marker genes reported in published literature for the corresponding cell types. Only concordant results between automated annotation and marker-based validation were retained for final cell type assignment.

## 3. Results

### 3.1. scRNA-Seq Identifies Distinct Cell Types in the Pituitary

**Figure 1:**
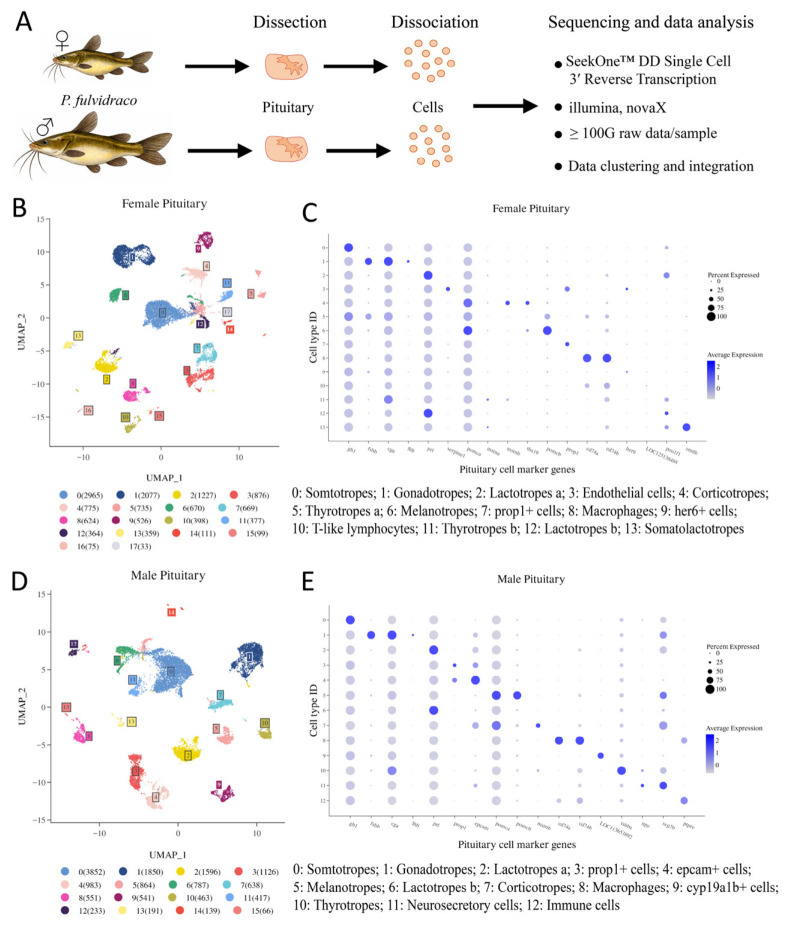
Single-cell transcriptomic profiling of female and male *P. fulvidraco* pituitaries prior to reproduction. (**A**) Schematic overview of the experimental workflow for single-cell RNA sequencing (scRNA-seq) of female and male pituitaries. (**B**,**D**) UMAP visualization of clustered pituitary cells, showing 17 distinct cell types in the female pituitary (**B**) and 15 cell types in the male pituitary (**D**). Each dot represents a single cell, colored according to its cluster identity. (**C**,**E**) Expression of canonical pituitary marker genes used to classify hormone-secreting endocrine cell types in females (**C**) and males (**E**).

To explore the transcriptomic landscape of pituitary cells in *P. fulvidraco* prior to reproduction, we performed single-cell RNA sequencing (scRNA-seq) on sexually mature female and male pituitaries (Figure 1A). Sequencing yielded a total of 361,532,970 and 423,620,826 raw reads for the female and male samples, respectively, with over 99.6% retained as high-quality clean reads. Following stringent quality control, 12,960 cells from the female pituitary and 14,297 cells from the male pituitary were retained for downstream analysis. The median number of genes detected per cell was 1688 in females and 1610 in males, indicating high transcriptome coverage and data quality.

We integrated the single-cell sequencing data from female and male pituitaries and performed clustering analysis, which revealed 17 distinct cell types in the female pituitary (Figure 1B) and 15 cell types in the male pituitary (Figure 1D). Using established pituitary marker genes as references [21], nine hormone-secreting endocrine cell types were identified in the female pituitary: somatotropes, gonadotropes, lactotropes a, corticotropes, thyrotropes a, melanotropes, thyrotropes b, lactotropes b, and somatolactotropes (Figure 1C). In the male pituitary, seven hormone-secreting endocrine cell types were classified: somatotropes, gonadotropes, lactotropes a, melanotropes, lactotropes b, corticotropes, and thyrotropes (Figure 1E). Notably, female pituitaries contained fewer somatotropes but more gonadotropes than male pituitaries (Table 1). Two distinct thyrotrope population (*tshba*^+^) were identified in the female pituitary, whereas only a single thyrotrope type was observed in the male pituitary (Figure 1C,E). The proportions of corticotrope and thyrotrope cells are higher in the female pituitary than in the male pituitary (Table 1).

### 3.2. Expression of Endocrine Regulatory Factor Receptor Genes in Pituitary Cells

**Figure 2:**
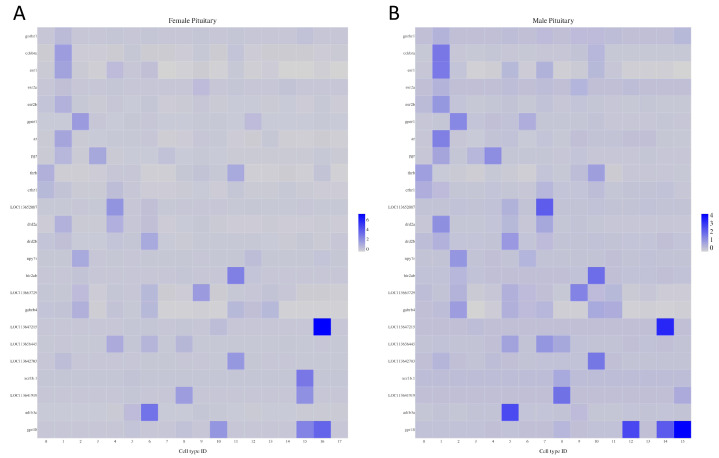
Cell-type-specific expression of receptor genes in female and male pituitaries. (**A**,**B**) Heatmaps showing expression of highly enriched receptor-encoding genes across pituitary cell types in females (**A**) and males (**B**).

To investigate cell-type-specific regulation of pituitary hormones, we analyzed receptor-encoding genes with high expression in pituitary cells. In total, 24 receptor-encoding genes were highly expressed in the pituitary. In the female pituitary (Figure 2A), somatotropes exhibited high expression of thyroid hormone receptor beta (*thrb*) and corticotropin-releasing hormone receptor 1 (*crhr1*). In gonadotropes, gonadotropin-releasing hormone receptor 1 (*gnrhr1*), cholecystokinin B receptor a (*cckbra*), estrogen receptor 1 (*esr1*), estrogen receptor 2b (*esr2b*), androgen receptor (*ar*), progesterone receptor (*pgr*), dopamine receptor D2a (*drd2a*), and dopamine receptor D2b (*drd2b*) were highly expressed. Lactotropes expressed G-protein-coupled estrogen receptor 1 (*gper1*), neuropeptide Y receptor Y7 (*npy7r*), and gamma-aminobutyric acid type A receptor subunit beta4 (*gabrb4*). In the male pituitary (Figure 2B), receptor gene expression patterns were largely similar to those in females. Somatotropes, gonadotropes, and lactotropes showed comparable expression profiles of the above-mentioned receptor genes. In the female pituitary, two thyrotrope subtypes were identified. Among them, only thyrotrope b cells resembled the thyroid-stimulating hormone-producing cells in the male pituitary, characterized by expression of the 5-hydroxytryptamine receptor 2A (*htr2ab*) and a metabotropic glutamate receptor 4-like gene (LOC113642703) (Figure 2A,B).

### 3.3. Ovarian Single-Cell Transcriptome Analysis

**Figure 3:**
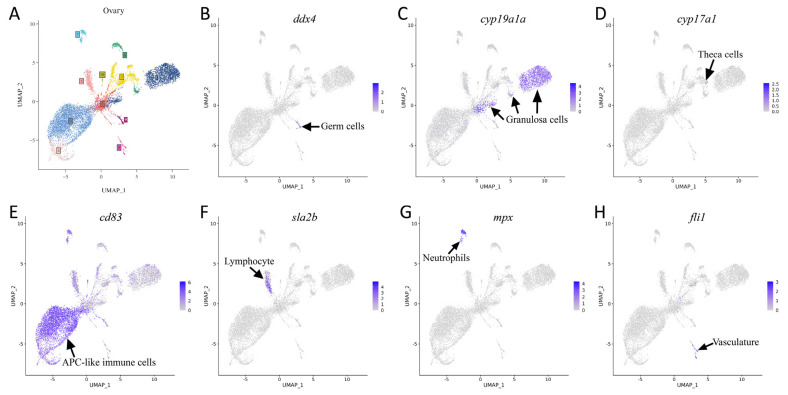
Single-cell transcriptomic profiling of pre-spawning ovaries. (**A**) UMAP visualization of 10 distinct ovarian cell types identified by scRNA-seq. Each dot represents a single cell, colored according to its cluster identity. (**B**–**H**) Expression of canonical marker genes in ovarian cell populations: germ cells marked by *ddx4* (**B**); granulosa cells by *cyp19a1a* (**C**); theca cells by *cyp17a1* (**D**); APC-like immune cells by *cd83* (**E**); lymphocytes by *sla2b* (**F**); neutrophils by *mpx* (**G**); and endothelial cells of the vasculature by *flil* (**H**).

Single-cell RNA sequencing of pre-spawning female *P. fulvidraco* ovaries identified a total of 10 distinct cell types (Figure 3A). Using well-established marker genes, seven major cell populations were annotated. *ddx4* expression specifically marked germ cells (Figure 3B), while *cyp19a1a* and *cyp17a1* were used to identify granulosa cells and theca cells (Figure 3C,D), respectively. The low proportion of germ cells is mainly due to the exclusion of large oocytes during tissue dissociation and filtration. Consequently, the recovered reproductive cell population was predominantly composed of oogonial stem cells and early-stage oocytes. In the ovary, approximately 22.9% of the cells were identified as granulosa cells and 8.6% as theca cells based on distinct transcriptional profiles. Among immune-related populations, *cd83* marked APC-like immune cells (Figure 3E), whereas *sla2b* and *mpx* were expressed in lymphocytes and neutrophils (Figure 3F,G), respectively. Endothelial cells of the vasculature were identified by *flil* expression (Figure 3H). The remaining three clusters were not definitively annotated.

**Figure 4:**
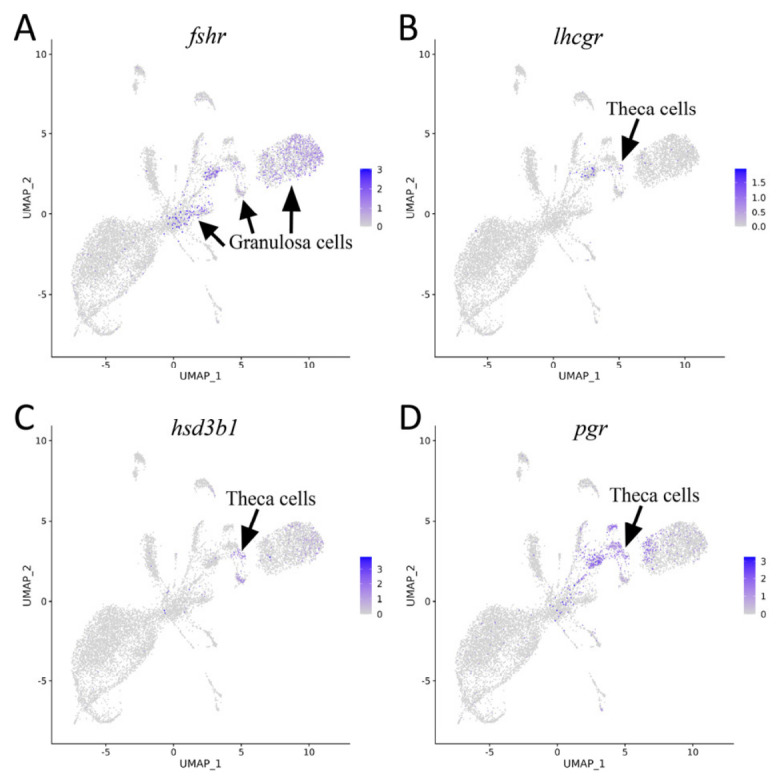
Cell-type-specific expression of gonadotropin receptors and steroidogenic pathway genes in the ovaries. (**A**,**B**) Expression of key gonadotropin receptor genes. Follicle-stimulating hormone receptor (*fshr*) was highly expressed in granulosa cells, with lower expression in theca cells (**A**). Luteinizing hormone receptor (*lhcgr*) was primarily expressed in theca cells, with a subset of granulosa cells showing detectable expression (**B**). (**C**) Steroidogenic pathway gene *hsd3b1* was predominantly expressed in theca cells, with lower expression in granulosa cells. (**D**) Progesterone receptor (*pgr*) was mainly expressed in theca cells.

Analysis of key gonadotropin receptor genes revealed distinct cell-type-specific expression patterns. The follicle-stimulating hormone receptor gene (*fshr*) was highly expressed in granulosa cells, with lower expression observed in theca cells (Figure 4A). In contrast, luteinizing hormone receptor (*lhcgr*) was primarily expressed in theca cells, with only a subset of granulosa cells showing detectable expression (Figure 4B). These results indicate that follicle-stimulating hormone (FSH) and luteinizing hormone (LH) act on different ovarian cell populations. Steroidogenic pathway genes also showed cell-type specificity. *hsd3b1* (hydroxy-delta-5-steroid dehydrogenase), a key enzyme in steroid hormone biosynthesis, was predominantly expressed in theca cells, while granulosa cells exhibited lower expression (Figure 4C). Similarly, *pgr* (progesterone receptor) was mainly expressed in theca cells, with relatively low expression in granulosa cells (Figure 4D). These findings suggest that theca cells are the primary site of steroidogenesis and progesterone signaling in the pre-spawning ovary.

### 3.4. Paracrine Regulation by Prostaglandin and Melatonin Signaling in the Ovaries

**Figure 5:**
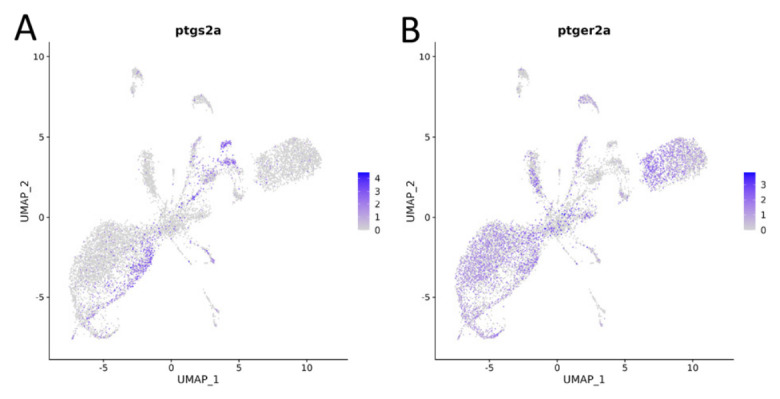
Expression of prostaglandin pathway genes in the ovary. (**A**) Expression of prostaglandin-endoperoxide synthase 2a (*ptgs2a*) in ovarian cell types. (**B**) Expression of prostaglandin E receptor 2a (*ptger2a*) across ovarian cells.

In addition to gonadotropin and steroid signaling, prostaglandin (PG) and melatonin (MT) pathways were implicated in ovarian regulation. The prostaglandin-synthesizing enzyme coding gene *ptgs2a* (prostaglandin-endoperoxide synthase 2a) was highly expressed in germ cells, theca cells, and immune cells, but showed minimal or no expression in granulosa cells (Figure 5A). Conversely, the prostaglandin receptor gene *ptger2a* (prostaglandin E receptor 2a) was enriched in granulosa cells (Figure 5B), suggesting that prostaglandins produced by theca cells act in a paracrine manner on granulosa cells to regulate oocyte maturation and ovulation.

**Figure 6:**
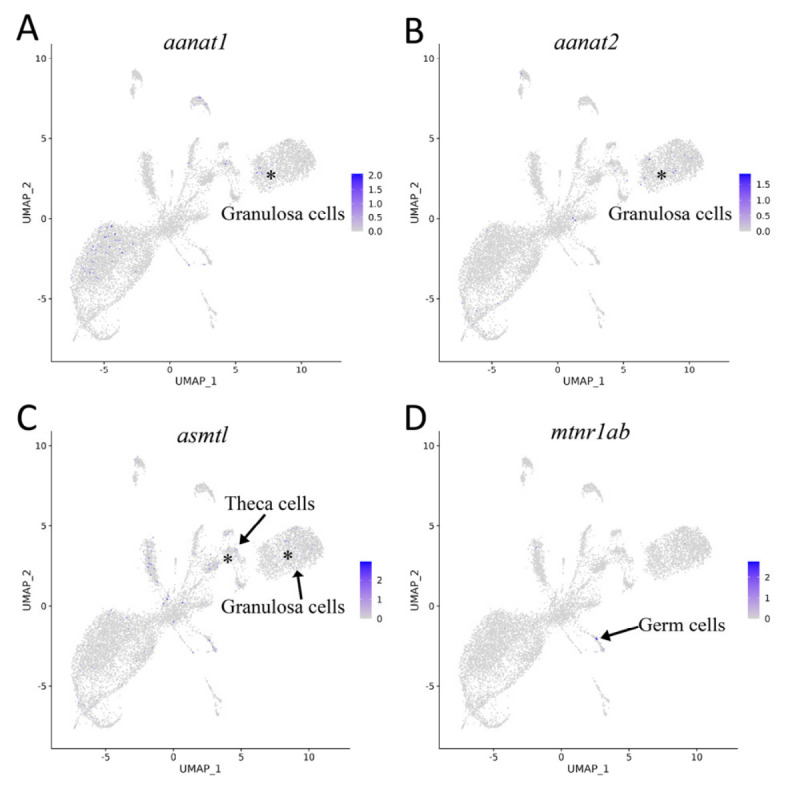
Expression of melatonin synthesis and receptor genes in the ovary. (**A**–**C**) Expression of melatonin synthesis genes *aanat1*, *aanat2*, and *asmtl* in ovarian cells, showing specific enrichment in granulosa cells. (**D**) Expression of the melatonin receptor gene *mtnr1ab*, which is predominantly detected in germ cells. * marks the location of cell distribution.

Melatonin synthesis genes, including *aanat1*, *aanat2*, and *asmtl*, were specifically expressed in granulosa cells (Figure 6A–C), whereas the melatonin receptor gene *mtnr1ab* was expressed in germ cells (Figure 6D). This indicates that ovarian cells produce melatonin that functions in a paracrine manner to modulate germ cell activity and reproductive processes.

## 4. Discussion

### 4.1. Pituitary Cell Heterogeneity and Hormone Regulation

The pituitary serves as the central endocrine organ coordinating reproduction, growth, and metabolism. Our scRNA-seq analysis identified 17 and 15 distinct cell types in the pituitaries of female and male *P. fulvidraco*, respectively, including multiple hormone-secreting endocrine populations. Female pituitaries contained fewer somatotropes but more gonadotropes than males, consistent with an increased demand for reproductive hormone secretion to support oocyte maturation [22,23]. The enrichment of somatotropes in the male pituitary of *P. fulvidraco* may contribute to the markedly higher growth rate of males relative to females. In females, two distinct thyrotrope populations (*tshba*^+^) were identified. Notably, the thyrotrope b cluster exhibited approximately 10-fold higher *tshba* expression than thyrotrope a. Thyrotropes b represent the primary cell population responsible for the synthesis and secretion of thyroid-stimulating hormone (TSH). In contrast, only a single *tshba*-expressing cell cluster was detected in males, and its expression level was approximately half of that observed in females. The observed sexual dimorphism in thyrotrope subtypes parallels previous reports that pituitary composition and endocrine responsiveness differ between sexes in teleost fish [24,25]. It suggests sex-specific specialization in thyroid hormone regulation, potentially coordinating metabolism and reproductive processes [26]. Furthermore, higher proportions of corticotrope and thyrotrope cells in females may reflect enhanced endocrine capacity to meet the energetic and stress-related demands of reproduction [24].

Expression patterns of receptor genes suggest that different pituitary cell types possess distinct regulatory capacities. Overall, endocrine receptor expression patterns in pituitary cell types appear largely conserved between males and females of *P. fulvidraco*. Somatotropes express receptors such as thyroid hormone receptor (*thrb*) and corticotropin releasing hormone receptor (*crhr1*), highlighting cross-talk between growth, metabolism, and reproductive axes [27]. Gonadotropes express receptors for hypothalamic and steroidal signals (e.g., *gnrhr1*, *esr1*/*2b*, *ar*, *pgr*), consistent with roles in integrating upstream reproductive cues [28]. The expression of *cckbra* was detected in gonadotropes, suggesting that hypothalamic or peripheral cholecystokinin (CCK) may act as a nutrient sensor involved in the regulation of gonadal development in teleosts [29,30]. We identified two thyrotrope subtypes (a and b) in the female pituitary. Receptor expression analysis revealed that thyrotrope b in females exhibits a transcriptional profile similar to male thyrotropes, including expression of *thrb*, *htr2ab*, *gabrb4*, and metabotropic glutamate receptor 4-like (LOC113642703). In contrast, female thyrotropes a showed only weak expression of the *adrb3a* receptor gene, suggesting potential functional specialization between the two subtypes. The expression of *adrb3a* in thyrotrope a suggests a potential involvement of β3-adrenergic signaling in the regulation of pituitary thyroid axis activity. Epinephrine/norepinephrine may activate the cAMP–PKA pathway in this cell population, thereby modulating *tshba* transcription and thyrotrope function [31]. This indicates that thyrotrope a may represent a stress-responsive regulatory subpopulation that indirectly influences thyroid hormone output and energy allocation during reproductive transitions.

### 4.2. Cellular Specialization in the Ovary

Our ovarian scRNA-seq analysis revealed 10 distinct cell populations including granulosa cells, theca cells, germ cells, and immune and vascular cells. The cell proportions observed are consistent with histological studies of teleost ovaries, where immune, granulosa and germ cells constitute the majority of ovarian mass [32]. The pronounced expression of *fshr* in granulosa cells and *lhcgr* in theca cells parallels previous characterizations of gonadotropin receptor localization in teleost follicles, where FSH primarily stimulates early follicle growth and LH promotes final maturation and ovulation [33]. Steroidogenic genes (*hsd3b1*) and progesterone receptor (*pgr*) were predominantly localized to theca cells, in agreement with findings that theca cells are the principal steroidogenic compartment in teleosts [34,35]. These results reinforce the concept of distinct endocrine roles for granulosa and theca cells during folliculogenesis.

### 4.3. Paracrine Roles of Prostaglandins and Melatonin

Our demonstration that prostaglandin synthesis gene *ptgs2a* is expressed in theca, germ, and immune cells, with the prostaglandin receptor *ptger2a* enriched in granulosa cells, supports a paracrine model of PG-mediated follicle regulation. This mechanism aligns with the well-established role of prostaglandins in fish ovulation, where PGs produced by follicular cells stimulate follicle rupture [36,37,38]. Melatonin synthesis genes (*aanat1/2*, *asmtl*) were enriched in granulosa cells, while melatonin receptors (*mtnr1ab*) were prominent in germ cells. These patterns suggest that locally synthesized melatonin acts in a paracrine manner to influence germ cell development, consistent with reports of melatonin’s involvement in seasonal reproductive regulation and oocyte maturation in fish [39,40,41]. Importantly, samples were collected in late March, a period characterized by progressively increasing photoperiod and rising ambient temperature in subtropical–temperate regions. In teleosts, increasing day length is generally associated with decreased systemic melatonin secretion but enhanced reproductive activation, indicating a potential shift from endocrine to local (ovarian) melatonin signaling.

### 4.4. Implications for Aquaculture and Future Research

The conserved organization of endocrine cell types in the pituitary is consistent with previous studies in teleost such as *Oryzias latipes* and *Danio rerio*, where classical endocrine lineages including gonadotropes, somatotropes, lactotropes, and thyrotropes have been identified at single-cell levels [17,25]. Similarly, reproductive endocrine regulation in Atlantic halibut is strongly dependent on gonadotropin-mediated control of ovarian follicle development, consistent with the granulosa- and theca-specific expression patterns observed in the present study [42]. Integrating cell composition and gene expression profiles highlights specific cellular targets for improving artificial breeding strategies. Granulosa and theca cells act as key integrators of endocrine and paracrine signals, offering potential biomarkers or manipulation targets for enhancing oocyte quality and spawning success [5,43]. Future work should investigate dynamic changes in these cell populations across reproductive stages and examine how environmental factors (e.g., photoperiod, temperature) modulate prostaglandin and melatonin signaling networks.

## 5. Conclusions

In conclusion, our single-cell atlas provides high-resolution insight into the cellular architecture and regulatory pathways governing pituitary and ovarian function in *P. fulvidraco*. Quantitative assessment of cell type proportions and signaling gene expression strengthens mechanistic understanding and supports translational applications in aquaculture.

## Figures and Tables

**Table 1:** Percent of hormone-secreting endocrine populations in female and male pituitaries.

Cell Types	Female	Male
Somatotropes	22.9%	26.9%
Gonadotropes	16.0%	12.9%
Lactotropes	12.3%	16.7%
Corticotropes	6.0%	4.5%
Thyrotropes	8.6%	3.2%
Melanotropes	5.2%	6.0%
Somatolactotropes	2.8%	2.9%

## Data Availability

The original data can be obtained by contacting the corresponding author.
